# HLA class I and II polymorphisms in pediatric neuroblastoma: mechanistic links to immune surveillance, tumor evolution, and clinical translation

**DOI:** 10.3389/fgene.2026.1918845

**Published:** 2026-10-21

**Authors:** Amira Daldoul, Wassim Y. Almawi

**Affiliations:** 1 Department of Oncology, Fattouma Bourguiba University Hospital, Monastir, Tunisia; 2 Faculty of Sciences, El-Manar University, Tunis, Tunisia

**Keywords:** antigen presentation, HLA, HLA class I, HLA class II, neuroblastoma, pediatric solid tumors

## Abstract

**Background:**

Pediatric solid tumors, including neuroblastoma (NB), emerge within an immature immune system where inherited immunogenetic variation may affect tumor onset and progression. Differences in HLA system are central to antigen presentation and immune recognition. However, their role in pediatric solid tumors is less clearly defined than in hematologic malignancies, and current evidence remains limited.

**Objective:**

This review summarizes evidence linking inherited HLA class I and II variation to NB susceptibility and clinical outcomes in pediatric NB. Tumor-associated HLA alterations, including reduced HLA expression and antigen-presentation defects that contribute to immune escape, are discussed as distinct but biologically related mechanisms.

**Methods:**

A structured synthesis of case–control and cohort studies evaluating HLA alleles, haplotypes, and expression in pediatric NB was performed. Data were organized by population, sample size, HLA locus, and association strength, and interpreted using concepts of antigen presentation, immune editing, and tumor microenvironment interactions.

**Results:**

Current evidence suggests that HLA-A, HLA-B, and HLA-C class I alleles and haplotypes influence susceptibility through effects on cytotoxic T-cell and natural killer cell function, while the strongest class II associations involve *DRB1-DQB1* region, including DQB1*05:02 and DRB1*16:01, which regulate CD4^+^ T-cell responses and inflammatory signaling. Some associations appeared subtype-specific, particularly in MYCN-amplified tumors, although evidence stratified by stage, age, and risk group remains limited. Beyond susceptibility, specific class I variants were associated with overall survival and progression-free survival including among patients receiving anti-GD2 immunotherapy, while HLA–KIR interactions and transplantation-related immunogenetic parameters were associated with disease progression and survival following hematopoietic stem cell transplantation. Separately, tumor-associated reductions in human leukocyte antigen expression or antigen-processing capacity may facilitate immune evasion by limiting effective antitumor immune recognition, though direct evidence remains limited.

**Conclusion:**

HLA polymorphisms, particularly within *HLA-A*, *HLA-B*, *HLA-C*, *DRB1*, and *DQB1* loci, are plausible, but not fully confirmed determinants of NB susceptibility and clinical outcome, with the strongest evidence relating to OS and PFS, clinical outcomes among patients receiving anti-GD2 immunotherapy, and outcomes following stem cell transplantation.

## 1 Introduction

Pediatric solid tumors constitute a diverse group of malignancies, including neuroblastoma (NB), Wilms tumor, hepatoblastoma, and pediatric sarcomas, accounting for roughly 40–45% of childhood cancers, with an incidence of about 150–160 cases per million children under 15 in developed countries (Allen-Rhoades et al., 2018; Belgiovine et al., 2024). These cancers arise within a developing immune system and differ fundamentally from adult malignancies in their genetic composition and evolutionary pathways (Sherif et al., 2022; Steliarova-Foucher et al., 2017). Unlike adult cancers, which are often linked to environmental exposures and accumulate multiple somatic mutations, pediatric tumors generally show low mutational burdens and are driven instead by developmental disruptions, recurrent chromosomal changes, and lineage-specific oncogenic programs (Bever and Song, 2023; Sherif et al., 2022). More than 60 tumor types occur across children, adolescents, and young adults, with approximately 8–15% associated with inherited cancer predisposition syndromes (Hughes et al., 2024; Steliarova-Foucher et al., 2017; Tonorezos and Yeh, 2026). Among these, NB is particularly informative for immunogenetic study, given its early developmental origin, frequent immune evasion, distinct molecular features such as MYCN amplification, and emerging links between HLA variation, disease susceptibility, and treatment response.

Genomic and transcriptomic advances have enabled refined molecular classification and the identification of key drivers of pediatric tumorigenesis (Zhang et al., 2024). However, tumor-intrinsic alterations explain only a subset of disease biology (Pavinato and Baggiolini, 2025; Zhang et al., 2024). Converging evidence highlights tumor–immune interactions as critical determinants of tumor initiation, progression, and therapeutic response in pediatric solid malignancies (Racacho et al., 2025). In this context, variation in the human leukocyte antigen (HLA) region is particularly relevant, given its central role in antigen presentation and immune recognition (Haworth et al., 2015; Shklovskaya and Rizos, 2021). The relatively low neoantigen burden characteristic of pediatric tumors suggests that inherited variation in antigen-presentation pathways may disproportionately influence susceptibility and clinical behaviour; nevertheless, these contributions remain insufficiently defined (Haworth et al., 2015; Maggi et al., 2025).

Two levels of HLA biology are relevant to pediatric tumors: germline HLA polymorphism, where inherited allelic/haplotypic variation influences antigen presentation, immune surveillance, and tumor susceptibility; and tumor-associated HLA alterations, including class I downregulation, loss of heterozygosity, and antigen-processing defects that arise during tumor evolution and promote immune evasion. While these processes may interact, they represent distinct biological mechanisms. Beyond their role in T-cell-mediated immunity, HLA class I molecules regulate natural killer (NK) cell function through interactions with killer-cell immunoglobulin-like receptors (KIRs), providing an important link between HLA genetics and innate antitumor immunity. This review adopts an NB-centered framework because NB is the most extensively characterized model among pediatric solid tumors for investigating inherited HLA variation, tumor HLA expression, natural killer (NK) cell interactions, and immunotherapeutic responses. It is also supported by the most comprehensive body of HLA genetic association data, reflecting both its relative prevalence among extracranial pediatric solid tumors and its distinctive pattern of HLA class I downregulation and immune evasion.

Among the classical loci studied, the most informative associations involve *HLA-A*, *HLA-B*, *HLA-C*, *DRB1*, and *DQB1*, which this review focuses on. Accordingly, NB serves as the principal context for examining the contributions of HLA class I and class II to susceptibility, immune surveillance, and clinical outcomes in pediatric solid tumors. Evidence from other tumor types is incorporated selectively where it clarifies broader immunogenetic mechanisms and informs the extent to which these findings may generalize across pediatric malignancies.

## 2 Methodology

### 2.1 Study design and scope

This narrative review provides a structured synthesis of studies evaluating associations between HLA class I and class II alleles or haplotypes and susceptibility, prognosis, and treatment response in pediatric NB, and was conducted through February 2026. Although literature identification followed predefined search and eligibility criteria, the review was not a formal systematic review and did not follow PRISMA methodology. Relevant literature was identified through structured searches of PubMed/MEDLINE and Scopus using combinations of the terms “HLA,” “human leukocyte antigen,” “MHC,” “neuroblastoma,” “polymorphism,” “genotype,” and “susceptibility,” supplemented by manual screening of reference lists from relevant articles. Most included studies were conducted in populations of European or North American ancestry, with limited representation of other ethnic groups. Given the substantial variation in HLA allele and haplotype frequencies across populations, this limited diversity represents an important constraint that may affect the generalizability of findings and reduce the capacity to detect population-specific associations.

Titles and abstracts were initially assessed for relevance, followed by full-text evaluation of potentially eligible articles. Studies were considered eligible when they reported original data on HLA class I or class II variation, expression, haplotypes, or related immunogenetic mechanisms in NB. Case–control, cohort, and genetic association studies published in English were considered. Studies limited to non-classical HLA loci without corresponding classical class I/II data, or restricted to non-pediatric populations, were excluded except where they were used for comparative context. We considered clinical and molecular stratification, including *MYCN* amplification, disease stage, risk group, age at diagnosis, and other genomic features. However, inconsistent reporting across studies limited direct comparison of subgroup-specific associations. We did not apply a formal PRISMA framework because the objective was a narrative synthesis rather than systematic evidence aggregation.

AD conducted the initial literature search and title/abstract assessment, and both authors reviewed potentially eligible full-text articles. Because the exact database-specific executable search strings were not prospectively archived, the principal search concepts and terms were reported to describe the search strategy. However, the complete search histories cannot be reconstructed retrospectively. Information on study population, ancestry, sample size, HLA locus or haplotype, typing methodology, NB subtype or clinical setting, effect estimates, statistical significance, and principal clinical or biological findings was extracted from the original publications and organized for narrative comparison. Attention was given to MYCN amplification, disease stage, risk group, age at diagnosis, treatment context, and evidence of independent replication, when available. Disagreements or uncertainties regarding interpretation were resolved through discussion between the authors.

### 2.2 Study size and statistical analysis

Most studies included in this review have modest sample sizes, reflecting both the rarity of NB and the challenges of assembling large cohorts. Sample sizes range from fewer than 50 cases in smaller studies to several hundred cases and controls in larger investigations (Abrahámová and Májský, 1988; Bonfiglio et al., 2024; Zhang et al., 2018). Consequently, many studies may have limited statistical power to detect modest HLA effects, particularly for rare alleles, extended haplotypes, and subgroup-specific associations defined by molecular or clinical characteristics. Across studies, allele and genotype frequencies were typically compared between cases and controls using chi-square or Fisher’s exact tests, and measures of association, including odds ratios (ORs) and relative risks (RRs), were reported to estimate effect size. Approaches to correction for multiple testing, however, varied considerably, which may influence the robustness and comparability of reported associations.

As this work is a narrative review rather than a systematic review or meta-analysis, no formal risk-of-bias assessment or pooled effect estimates were undertaken. Instead, findings were interpreted in the context of study design, cohort size, HLA typing resolution, ancestry composition, and the application of multiple-testing corrections, as well as the extent of replication and availability of supporting mechanistic data. Emphasis was therefore placed on consistency of observed associations, biological plausibility, typing resolution, ancestry matching, and appropriate statistical correction, rather than on a formal hierarchy of evidence.

## 3 Pediatric neuroblastoma as an immunogenic model

### 3.1 Pathogenesis and pathology of pediatric neuroblastoma

Neuroblastoma, the most common extracranial malignancy of early childhood, originates from embryonic neural crest–derived precursors of the sympathetic nervous system and typically arises in the adrenal medulla or paraspinal ganglia (Campbell et al., 2024; Wang et al., 2020). It is most frequently diagnosed during infancy, with a median age at diagnosis of approximately 18 months, and nearly 90% of cases occurring before 10 years of age. Although relatively rare, with an age-standardized incidence of 5.5–11.5 cases per million in North America, NB accounts for a disproportionate fraction of pediatric cancer mortality (Filho et al., 2025). Clinical behavior is highly heterogeneous, ranging from spontaneously regressing localized tumors in infants to aggressive metastatic disease in older children (Campbell et al., 2024; Sainero-Alcolado et al., 2024). This variability is reflected in marked differences in survival across risk groups and persists despite advances in multimodal therapy (Campbell et al., 2024; Filho et al., 2025).

The biological and clinical heterogeneity of neuroblastoma (NB) is driven by distinct molecular features, including MYCN amplification and recurrent segmental chromosomal alterations such as deletions of 1p and 11q (Jiang et al., 2025; Wang et al., 2020), which are consistently associated with high-risk disease and adverse outcomes (Stöber et al., 2024; Wang et al., 2020). These variables are incorporated into the International Neuroblastoma Risk Group classification system, which integrates imaging-defined stage with clinical and genomic parameters to stratify patients (Monclair et al., 2009).

In parallel, NB displays immunobiological properties that distinguish it from most adult malignancies, including a low somatic mutational burden, reduced expression of classical HLA class I molecules, and expression of immunosuppressive non-classical and soluble HLA forms (Sainero-Alcolado et al., 2024; Seo et al., 2024). Together, these features contribute to an immunologically restricted tumor microenvironment and underscore the potential relevance of host HLA variation in modulating disease susceptibility and immune evasion (Airoldi et al., 2026; Maggi et al., 2025). Within this framework, the HLA system represents a central component of host–tumor interactions and immune escape mechanisms in NB (Bonfiglio et al., 2024; Lim et al., 2024; Shklovskaya and Rizos, 2021). Notably, HLA class I downregulation varies across NB subtypes, with MYCN-amplified tumors exhibiting more pronounced loss of classical HLA class I expression than non-amplified tumors (Sainero-Alcolado et al., 2024; Seo et al., 2024), highlighting subtype-specific differences that parallel the HLA haplotype associations discussed below.

### 3.2 The pediatric immune system

The pediatric immune system differs fundamentally from that of adults and exhibits developmental features directly relevant to tumor immunology and immune surveillance (Brodsky et al., 2025; Chen et al., 2025). In early life, naïve T and B lymphocytes predominate, with lower frequencies of memory and effector subsets, reflecting ongoing thymic selection and maturation of the T-cell repertoire (Adkins et al., 2004; Kollmann et al., 2017; Zhang et al., 2017). CD8^+^ T cells display a rapidly induced but short-lived effector phenotype, with innate-like characteristics, including elevated cytotoxic mediator expression and increased susceptibility to activation-induced cell death (Brodsky et al., 2025).

Innate immunity also demonstrates developmental immaturity, as pediatric natural killer cells exhibit reduced cytotoxic capacity along with distinct receptor expression patterns and cytokine profiles compared with adults (Brauning et al., 2022; Sundström et al., 2007). In addition, the CD4^+^/CD8^+^ T-cell ratio is elevated in infancy and gradually declines toward adult levels with age (Ge et al., 2025). Collectively, these features define an immunological milieu characterized by attenuated pro-inflammatory responses and evolving antigen-presentation capacity, which may influence tumor–immune interactions and modulate immune recognition and control of emerging malignancies (Maggi et al., 2025; Yu et al., 2024).

### 3.3 Structure and relevance of the HLA system in pediatric solid tumors

Located within the major histocompatibility complex (MHC) on chromosome 6p21.3–22.1 and spanning approximately 4 Mb, the HLA region is among the most gene-dense and polymorphic segments of the human genome (Sanchez-Mazas and Nunes, 2024; Turganbekova et al., 2025). It includes classical class I genes (HLA-A, -B, -C) and class II genes (DRA, DRB1/3/4/5, DQA1, DQB1, DPA1, DPB1) (Kang et al., 2023), as well as non-classical loci (HLA-E, -F, -G) and genes involved in antigen processing, complement activation, inflammation, and innate immune responses (Kang et al., 2023; Sanchez-Mazas and Nunes, 2024) (Figure 1). The extensive allelic diversity of this region underlies interindividual variation in immune recognition and responsiveness (Pagliuca et al., 2022; Sanchez-Mazas and Nunes, 2024), reflects population evolutionary history (Almawi et al., 2004; Hajjej et al., 2006), and contributes to susceptibility to infections, autoimmune diseases, and malignancies (Bonfiglio et al., 2024; Dorak et al., 1999; Fung and Pio, 2025). At the cellular level, HLA alleles shape T-cell repertoire diversity through peptide–HLA–TCR interactions that regulate selection, expansion, and differentiation (Fung and Pio, 2025; Lau et al., 2011; Lim et al., 2024).

**FIGURE 1:**
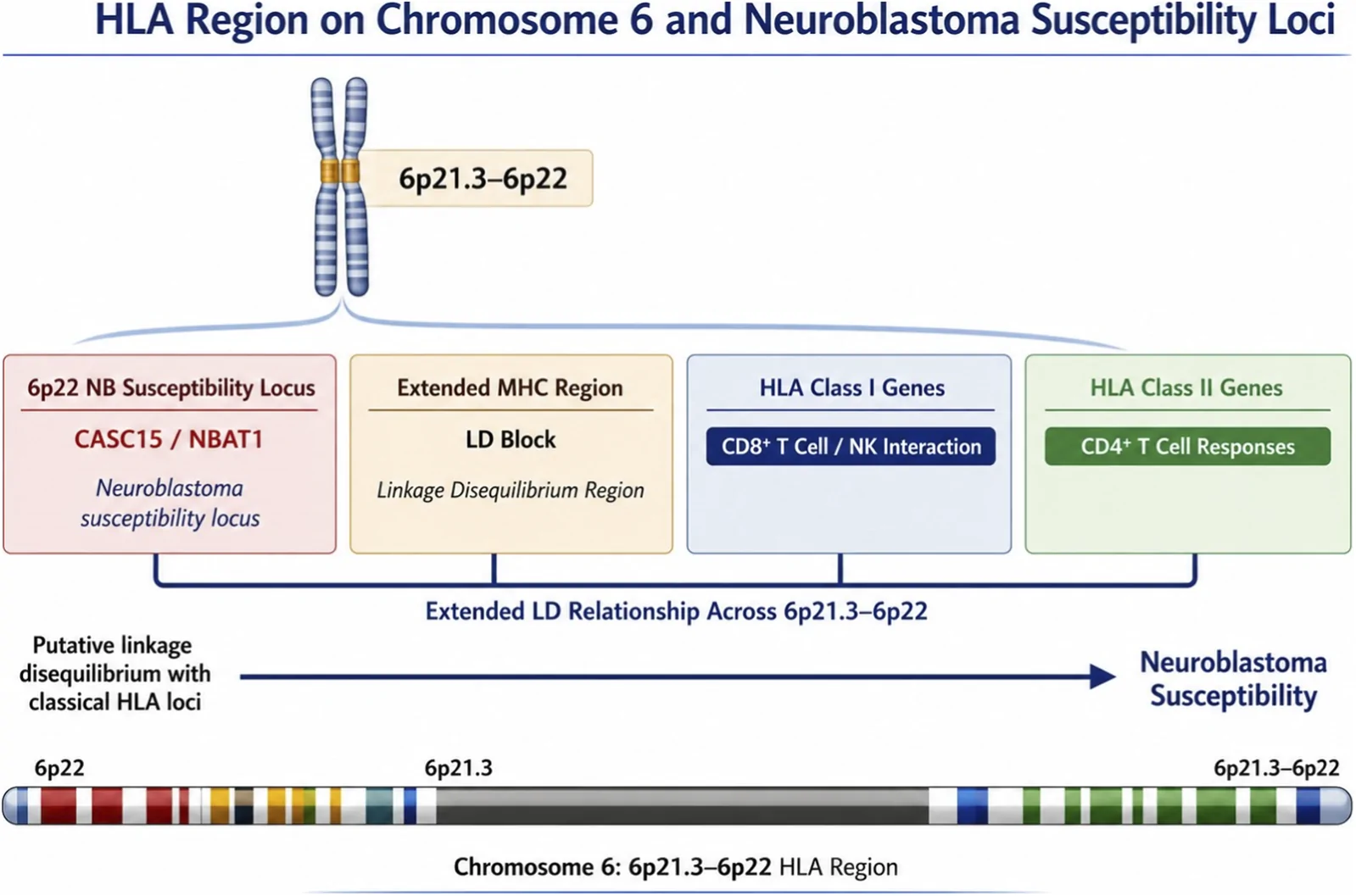
HLA region on chromosome 6 and its relationship to NB susceptibility loci. The classical HLA class I and class II regions within chromosome 6p21.3 in close proximity to the adjacent 6p22 CASC15/NBAT1 NB susceptibility locus. Because this genomic segment exhibits substantial LD across 6p21.3-6p22, susceptibility variants near CASC15/NBAT1 can be co-inherited with specific HLA alleles or haplotypes, complicating interpretation of association signals. The relationship highlighted here underscores the importance of distinguishing replicated GWAS signals arising from 6p22 from direct effects attributable to classical HLA variation, which instead require dedicated HLA typing and conditional analytical approaches to resolve. Created with BioRender.com and Adobe Illustrator.

In malignancy, inherited HLA polymorphisms influence peptide presentation and cancer susceptibility, whereas acquired tumor-associated alterations, including HLA class I loss, loss of heterozygosity, and antigen-processing defects, impair CD8^+^ T-cell recognition and promote immune escape. In addition, reduced or absent HLA class I expression impairs CD8^+^ T-cell–mediated tumor recognition while altering susceptibility to natural killer (NK) cell cytotoxicity (Haworth et al., 2015; Shklovskaya and Rizos, 2021). HLA class II–restricted pathways further influence clinical outcomes by modulating CD4^+^ T-cell activation, cytokine signaling, tumor-associated inflammation, and interactions with NK-cell receptors (Baretti et al., 2025; Delgado et al., 2010; Erbe et al., 2018; Wang et al., 2020). Although HLA polymorphism is well characterized in adult cancers and pediatric hematologic malignancies (Burkitbayev et al., 2026; Turganbekova et al., 2026), its role in pediatric solid tumors remains less well defined (Bonfiglio et al., 2024; Trubicka et al., 2022; UK Childhood Cancer Study Investigators, 2000). Recent studies report HLA loss of heterozygosity and reduced expression of antigen-processing components, both associated with immune evasion (Jiang et al., 2023; Lim et al., 2024), alongside links between specific class II variants and KIR–HLA interactions and disease susceptibility or treatment response (Erbe et al., 2018; Forlenza et al., 2016; Zhang et al., 2018).

## 4 HLA link to neuroblastoma

### 4.1 GWAS and the extended MHC region

The first genome-wide association study (GWAS) of NB, reported in 2008, identified chromosome 6p22 as a susceptibility locus (Maris et al., 2008). Three variants: rs6939340, rs4712653, and rs9295536, reached genome-wide significance and were subsequently replicated in independent cohorts, including Asian (Zhang et al., 2017) and non-Asian populations (Capasso et al., 2009), providing independent support for the 6p22 association. Additional evaluation in southern Chinese and Italian pediatric cohorts provided cross-population support for the 6p22 association (He et al., 2017). Homozygosity for the risk alleles was associated with *MYCN* amplification, stage 4 disease, and high-risk classification, indicating that this locus contributes to both tumor initiation and aggressive clinical behavior. The 6p22 signal maps telomeric to the classical HLA region (6p21.3), within the extended major histocompatibility complex (MHC) haplotype (Haworth et al., 2015). Fine-mapping studies localized the association to a 34.9 kb linkage disequilibrium (LD) block encompassing the long non-coding RNA genes *CASC15* and *NBAT1*, neither of which encodes classical HLA molecules (Mondal et al., 2018).

Despite this refinement, the localization of the associated block within the extended MHC suggests that linkage disequilibrium with adjacent classical HLA loci—particularly class I genes at 6p21.3—may contribute to the observed signal (Moutsianas et al., 2011). This potential confounding remains unresolved in NB, as conditional analyses and targeted HLA fine mapping are limited. Accordingly, the independent contribution of classical HLA alleles to NB susceptibility has not yet been established. Replication in southern Chinese and Italian pediatric cohorts corroborated the 6p22 association, with the rs6939340 AA genotype showing a protective effect in Chinese patients (He et al., 2017). Overall, these findings place the 6p22 locus near an immunogenetic region but do not provide direct evidence of a classical HLA association in the absence of dedicated HLA-focused analyses in NB cohorts.

Beyond 6p22, classical HLA class I and II loci within the MHC have been directly evaluated for association with NB risk (Figure 1). Although HLA polymorphism is increasingly implicated in tumor immunology in pediatric solid tumors, it has been less extensively characterized than in hematologic malignancies, with available studies constrained by small sample sizes, population-specific allele distributions, and heterogeneous genotyping approaches (Abrahámová and Májský, 1988; Fung and Pio, 2025). NB is a pertinent model in this context, as it arises during a period of immune maturation and exhibits mechanisms of immune evasion, including downregulation of HLA class I molecules and impairment of antigen-processing pathways (Verhoeven et al., 2022). Immunohistochemical and immunofluorescence analyses have reported reduced or absent HLA class I expression in subsets of NB tumors, although the frequency and extent of expression vary according to methodology, tumor subtype, and clinical setting. HLA class II expression has likewise been reported as low or undetectable in NB samples and evaluated experimental settings (Airoldi et al., 2026; Dhatchinamoorthy et al., 2021; Pabla et al., 2024). This should not be interpreted as demonstrating universal absence of HLA class II expression across all NB tumors, biological subtypes, or experimental conditions. Collectively, germline variation at individual HLA loci, particularly *HLA-B*, *HLA-C*, *HLA-A*, and *DRB1∼DQB1* and an extended *HLA-A ∼ B ∼ C* haplotype, may influence NB susceptibility and tumor–host interactions, whereas acquired HLA alterations represent distinct mechanisms of immune evasion. As only a subset of studies examined associations according to molecular subtypes or clinical risk characteristics, most notably *MYCN* amplification status, caution should be exercised when interpreting these findings.

### 4.2 Class I HLA variation and risk-modifying haplotypes

Studies of varying design and cohort size have examined the association between HLA class I variation and NB, including susceptibility, prognosis, and treatment response (Table 1), with inconsistent evidence for a generalized effect on disease risk. Of the classical class I genes, most reported associations involve *HLA-B* and *HLA-C* alleles, although an extended A*02:01∼B*44:02∼C*05:01 haplotype has shown the strongest enrichment in *MYCN*-amplified NB. The strongest high-resolution associations involve extended haplotypes and subtype-specific allele frequency differences, rather than individual alleles alone (Lim et al., 2024). However, these findings are based mainly on the MOSCATO-01/MAPPYACTS cohort and remain unvalidated in independent NB populations. Early serological analyses suggested a potential association between HLA-B13 and increased NB risk; however, this observation did not remain significant after correction for multiple testing and was limited by small sample size (Abrahámová and Májský, 1988). More recent large-scale analyses have not confirmed broad associations. In a European ancestry case–control study of 803 NB cases and 1,340 controls, no significant associations were detected for classical or non-classical HLA class I loci, including HLA-A, -B, -C, -E, -F, and -G (Bonfiglio et al., 2024). Similarly, an Australian study found no evidence linking HLA-G expression to NB susceptibility in 163 patients and 404 controls (Lau et al., 2011).

**TABLE 1:** HLA class I associations with neuroblastoma.

HLA Marker/Haplotype	Association	Ethnicity/Population	Sample Size (Case/Control)	Neuroblastoma Subtype	Magnitude of Association	Clinical Interpretation	References
A*02:01∼C*05:01∼B*44:02	Overrepresented	European pediatric cohort MOSCATO-01/MAPPYACTS	Neuroblastoma subset of 576 pediatric solid tumors	MYCN-amplified NB	Frequency = 0.143; relative abundance = 5.7	Enriched in MYCN-amplified NB	Lim et al., 2024
B*51:01	Overrepresented	Predominantly european pediatric cohort	Neuroblastoma subset of 576 pediatric solid tumors	Mixed NB	Relative abundance >1	Enriched among NB cases	
B*18:01							
C*12:03							
B*07:02	Underrepresented			MYCN-amplified and non-amplified NB	Relative abundance 0.31–0.58	Underrepresented among NB cases	
B*08:01				Mixed NB	Relative abundance <1	Underrepresented among NB cases	
C*07:02							
A*02:01	Favorable prognosis	Multi-institutional cohort	Not reported	All stages	HR = 0.72; p = 0.03	Improved overall survival	Fung and Pio, 2025
C*07:01					HR = 0.71; p = 0.03		
*HLA-Bw4-80I*	Adverse prognosis	North american/European cohort	245 patients	High-risk NB	OS HR = 2.23 (95% CI, 1.34–3.71); PFS HR = 2.43 (95% CI, 1.53–3.87)	Poorer response to anti-GD2 therapy	Forlenza et al., 2016
HLA-B13	Increased risk	Not specified	20 patients/301 controls	All stages	Non-significant after correction	Nominal association with increased NB odds	Abrahámová and Májský, 1988
HLA-G	No association	Not specified	163 patients/404 controls	Mixed NB	Not significant	No association	Lau et al., 2011
HLA-A, -B, -C, -E, -F, -G	No association	European ancestry	803 patients/1,340 controls	Mixed NB	Not significant	No association	Bonfiglio et al., 2024

Abbreviations: NB, neuroblastoma; MYCN, MYCN oncogene; HR, hazard ratio; OS, overall survival; PFS, progression-free survival. Associations reported in the table reflect findings from individual studies and should be interpreted in the context of study design, sample size, and statistical correction methods used by the original investigators.

Lim et al. conducted the most comprehensive evaluation to date, analyzing high-resolution HLA genotypes from predominantly European pediatric patients enrolled in the MOSCATO-01 and MAPPYACTS trials (Lim et al., 2024). Within the NB subset, the extended haplotype A*02:01∼B*44:02∼C*05:01 showed the strongest enrichment among affected individuals, particularly in MYCN-amplified tumors, a molecular subtype associated with poor prognosis (Lim et al., 2024). MYCN amplification has been linked to reduced HLA class I expression in NB, thereby diminishing recognition by cytotoxic T cells and facilitating immune evasion (Qiu and Matthay, 2022). Notably, associations differed by molecular subtype. The A*02:01∼B*44:02∼C*05:01 haplotype was most enriched in *MYCN*-amplified tumors, whereas B*07:02 depletion occurred in both amplified and non-amplified subgroups. These findings suggest subtype-specific inherited HLA effects that may influence CD8^+^ T-cell surveillance during tumorigenesis, although this mechanism remains unproven in NB.

Additional alleles enriched among NB cases in this cohort included B*51:01, B*18:01, and C*12:03 (Lim et al., 2024). In contrast, B*07:02, B*08:01, and C*07:02 were underrepresented among NB patients (Lim et al., 2024). Within the Lim et al. cohort, B*07:02 was depleted in both *MYCN*-amplified and non-amplified tumor subgroups, suggesting an association across NB subtypes. More effective immune recognition of transformed cells remains a biologically plausible explanation, although it was not functionally demonstrated in NB. The consistent depletion of B*07:02 across MYCN-amplified and non-amplified tumors indicates that this effect may extend across molecular subtypes (Lim et al., 2024). Beyond susceptibility, HLA class I variation has been linked to post-diagnosis clinical outcomes, including overall (OS) and progression-free survival (PFS). HLA-A*02:01 and C*07:01 have been independently associated with improved OS, consistent with enhanced antitumor immunity (Fung and Pio, 2025). In contrast, HLA-Bw4-80I has been associated with inferior OS and PFS in high-risk NB patients treated with anti-GD2 immunotherapy (Forlenza et al., 2016).

Beyond antigen presentation to CD8^+^ T cells, HLA class I molecules also regulate NK-cell activity, in which NK cells express inhibitory and activating KIRs that recognize specific HLA class I ligands (Chen et al., 2024). While engagement of inhibitory KIRs by self-HLA class I molecules normally suppresses NK-cell cytotoxicity (Chen et al., 2024; Draghi et al., 2005), reduced HLA class I expression can diminish inhibitory signaling and promote NK-cell activation through the ‘missing-self’ response (Duygu et al., 2021). In this context, it was shown that HLA-Bw4 molecules act as ligands for inhibitory KIRs expressed on NK cells, thereby modulating NK-cell activation (Delgado et al., 2010; Erbe et al., 2018; Forlenza et al., 2016), and engagement of KIRs by HLA-Bw4 transmits inhibitory signals that reduce NK-cell cytotoxicity (Kim et al., 2008; Verheyden et al., 2009). Because anti-GD2 immunotherapy relies primarily on NK cell–mediated antibody-dependent cellular cytotoxicity (Mise et al., 2016), stronger inhibitory HLA–KIR interactions may reduce therapeutic efficacy (Pollock et al., 2022; Verheyden et al., 2009). Conversely, the absence of specific inhibitory ligands may enhance NK-cell activation and antitumor responses (Hiura et al., 2025), highlighting HLA–KIR interactions as key determinants of treatment response in NB (Mise et al., 2016; Pollock et al., 2022; Verheyden et al., 2009), and that variation in HLA class I alleles may influence antitumor immunity through both adaptive immune responses and NK-cell-mediated surveillance (Chen et al., 2024).

Consistent with this framework, high-risk NB patients lacking the HLA-C1 ligand group exhibited reduced disease progression and improved survival following autologous hematopoietic stem cell transplantation (HSCT), likely reflecting enhanced NK-cell activation through “missing-self” recognition (Venstrom et al., 2009). More recently, higher PIRCHE-I/II mismatch scores have been associated with reduced progression and improved survival among relapsed NB patients undergoing haploidentical stem cell transplantation, suggesting that indirect T-cell recognition contributes to graft-versus-tumor effects (Seo et al., 2025). Collectively, these studies identify distinct levels of HLA-related information in NB. Germline HLA alleles and haplotypes are mainly associated with case status, subtype, and outcome; HLA ligand groups and HLA–KIR combinations reflect NK-cell regulation; and transplantation studies assess recipient or donor–recipient immunogenetic factors. As they represent different biological mechanisms and clinical contexts, these categories should be interpreted separately.

### 4.3 Class II HLA associations in neuroblastoma

Compared with HLA class I, the contribution of HLA class II variation to NB has been less extensively investigated. Among the available class II analyses, associations were reported within *DRB1–DQB1* region, particularly DRB1*16:01, DQB1*05:02, and the under-represented DRB1*15:01∼DQA1*01:02∼DQB1*06:02 haplotype. Among reported genetic associations, signals mapping to the class II region, particularly the *DRB1* locus, are among the most consistent and statistically robust (Table 2), supporting a role for class II–restricted antigen presentation and CD4^+^ T-cell–mediated immunity in NB pathogenesis (Bonfiglio et al., 2024; Couture et al., 2019; Lim et al., 2024). Because HLA class II molecules present peptides derived from extracellular sources to helper T cells, inherited variation at these loci may influence both the initiation and the effectiveness of antitumor immune surveillance during early malignant transformation (Seliger et al., 2017). However, despite this biological rationale, the available evidence remains limited.

**TABLE 2:** HLA class II associations with neuroblastoma^1^.

HLA Marker/Haplotype^2^	Association	Ethnicity/Population	Sample Size (Cases/Controls)	Neuroblastoma Subtype	Magnitude of Association	Clinical Interpretation	References
DRB1*15:01∼DQA1*01:02∼ DQB1*06:02	Underrepresented	Predominantly european pediatric cohort	Neuroblastoma subset of 576 pediatric solid tumors	Multiple NB subtypes	Frequency = 0.038–0.073; relative abundance = 0.31–0.58	Underrepresented among NB cases	Lim et al., 2024
DQB1*05:02	Increased frequency	European ancestry	724/2863	All NB	OR = 1.61; adj p = 5.4 × 10^−3^; OR = 1.606 (95% CI 1.273–2.018)	Enriched among NB cases	Bonfiglio et al., 2024
DRB1*16:01	Increased frequency	European ancestry	724/2863	All NB	OR = 1.60; adj p = 2.3 × 10^−2^	Enriched among NB cases	Bonfiglio et al., 2024
*DQB1 Ser57*	Risk-associated residue	European ancestry	724/2863	All NB	OR = 1.606 (95% CI, 1.273–2.018), p = 5.49 × 10^−5^, FDR-adjusted p = 2.19 × 10^−4^	Residue associated with the NB signal in conditional analysis	Bonfiglio et al., 2024
Composite HLA risk score	Adverse prognosis	European ancestry	724 patients	All NB	HR = 1.53; p = 0.032	Associated with overall survival in multivariable analysis	Bonfiglio et al., 2024

1 Interpreted within extended MHC (6p21.3)–6p22 context; LD may link HLA haplotypes with nearby NB susceptibility variants.

2 Includes allele- and residue-level signals; some reflect conditional analyses resolving independent HLA effects.

Early studies did not include HLA class II loci in their analyses (Abrahámová & Májský et al., 1988), and only a small number of investigations have specifically addressed their role in NB susceptibility. A recent large whole-exome sequencing study detected no associations between NB risk and class II genes (*DRB1*, *DQA1*, *DQB1*, *DPA1*, *DPB1*) at genome-wide significance (Bonfiglio et al., 2024). A subsequent locus-focused analysis of the same underlying dataset identified DQB1*05:02 and DRB1*16:01 as independent susceptibility alleles, each associated with an approximately 60% increase in the odds of NB in a cohort of 724 cases and 2,863 ancestry-matched controls (Bonfiglio et al., 2024). This effect was primarily attributed to DQB1*05:02 and the encoded Ser57 residue within the peptide-binding groove of the HLA-DQ molecule, suggesting that structural variation affecting peptide binding may alter immune recognition of transformed neural crest-derived cells (Bonfiglio et al., 2024). Because these findings derive from re-analysis of the same cohort rather than an independent replication cohort, their reproducibility remains to be established.

Haplotype-based analyses further support this interpretation. The extended haplotype DRB1*15:01∼DQA1*01:02∼DQB1*06:02 is consistently underrepresented across NB subgroups, including both MYCN-amplified and non-amplified tumors, suggesting a potential protective effect (Lim et al., 2024). Because this haplotype is common in European populations (Sanchez-Mazas and Nunes, 2024), its depletion in NB cases suggests that specific class II configurations may favor more effective immune surveillance. Enhanced recognition and elimination of premalignant cells represent a biologically plausible mechanism (Seliger et al., 2017), but has not been directly demonstrated for this haplotype in NB. These observations underscore the importance of extended haplotype analysis, as functional effects within the HLA region often reflect combinations of tightly linked loci rather than single alleles. Furthermore, class II associations appear to be context-dependent, requiring a compatible T-cell receptor repertoire and coordinated adaptive immune activation to produce measurable effects (Fung and Pio, 2025).

In contrast to many genetic factors linked to aggressive disease features, the currently identified HLA class II variants appear to primarily influence susceptibility rather than disease progression (Lim et al., 2024; Pavlick et al., 2024). Although DQB1*05:02 and DRB1*16:01 increase the likelihood of NB development, they have not consistently been linked to adverse molecular characteristics or more aggressive clinical phenotypes after disease onset (Bonfiglio et al., 2024). Nevertheless, a composite HLA genetic risk score has been associated with reduced OS, indicating that host immunogenetic background may continue to shape tumor–immune interactions during disease progression (Bonfiglio et al., 2024). Collectively, these data associate variation within the HLA class II region, particularly the *DRB1–DQB1* interval, with NB susceptibility. Modulation of antigen presentation and CD4^+^ T-cell–dependent immune responses provides a biologically plausible explanation for these associations, but these associations remain to be functionally demonstrated in NB.

### 4.4 HLA-KIR interactions and NK-Cell surveillance in neuroblastoma

NK cells are particularly relevant to NB, given its low mutational burden and variable HLA class I expression, which may limit conventional T-cell-mediated tumor recognition. Consequently, NK-cell cytotoxicity represents an important component of antitumor immunity (Delgado et al., 2010). Interactions between individual HLA class I ligands and killer-cell immunoglobulin-like receptors (KIRs) regulate NK-cell activation thresholds, suggesting that inherited HLA–KIR combinations may influence immune surveillance and tumor–host interactions (Hiura et al., 2025; Venstrom et al., 2009). Conversely, acquired alterations in tumor HLA class I expression may shift the balance between T-cell recognition and NK-cell activation, with reduced HLA expression potentially impairing cytotoxic T-cell recognition while diminishing inhibitory KIR signaling and enhancing NK-cell cytotoxicity (Erbe et al., 2018; Verheyden et al., 2009). Although these mechanisms are well established in NK-cell biology, they remain unproven in the HLA-KIR associations reported in NB and should be considered a mechanistic hypothesis.

This axis may also influence anti-GD2 immunotherapy, in which NK-cell-mediated antibody-dependent cellular cytotoxicity contributes substantially to therapeutic efficacy (Forlenza et al., 2016; Verheyden et al., 2009). Thus, HLA-associated effects may extend beyond antigen presentation to innate immune regulation and may be associated with clinical outcomes during anti-GD2 immunotherapy (Delgado et al., 2010; Hiura et al., 2025), although integrated HLA–KIR studies in NB remain limited, and a treatment-specific predictive effect has not been established.

## 5 Discussion

### 5.1 Integrated class I/Class II model

The principal class I loci implicated in NB across reviewed studies include *HLA-A*, *HLA-B*, and *HLA-C*, with the strongest signal arising from the extended A*02:01∼B*44:02∼C*05:01 haplotype and recurrent effects involving HLA-B alleles. By contrast, the principal reported class II associations involve the *DRB1-DQB1* region, particularly DQB1*05:02 and DRB1*16:01, although these findings lack independent replication. Taken together, the available evidence indicates that variation in both HLA class I and class II loci contribute to NB susceptibility, with the strongest reported associations occurring at the level of extended haplotypes rather than individual alleles. For class I, HLA-A*02:01∼B*44:02∼C*05:01 haplotype is enriched, whereas B*07:02 is consistently depleted across *MYCN*-amplified and non-amplified tumors, suggesting an association consistent with, but does not establish, differential CD8^+^ T-cell–mediated immune surveillance during tumorigenesis (Lim et al., 2024). For class II, DQB1*05:02 and DRB1*16:01 are independently linked with increased risk, with DQB1*05:02-encoded Ser57 residue linking peptide-binding groove variation to altered immune recognition of transformed neural crest-derived cells (Bonfiglio et al., 2024). In contrast, DRB1*15:01∼DQA1*01:02∼DQB1*06:02 haplotype is consistently underrepresented among NB cases (Lim et al., 2024), consistent with an association with decreased odds of NB. However, interpretation must account for NB’s biological heterogeneity: *MYCN* amplification reveals subtype-specific haplotype and allele patterns, while stage, risk group, age, and other features are rarely reported, underscoring the need for standardized stratification.

The literature points at two distinct facets of HLA biology: germline HLA polymorphisms influencing immune responsiveness, antigen presentation, and tumor susceptibility, and acquired tumor-associated alterations, including HLA loss, loss of heterozygosity, and impaired antigen processing, linked with reduced tumor immune visibility and promotion of immune escape (Figure 2). These findings partially address the question raised by the chromosome 6p22 GWAS signal (see *HLA Link to Neuroblastoma*, above): although the 6p22 locus itself does not encode classical HLA molecules, the independent associations identified directly within the class I and class II regions support the possibility that classical HLA variation contributes to NB susceptibility independently of, or alongside, the 6p22/CASC15–NBAT1 signal; however, this remains to be tested formally using conditional models. The pattern of subtype-specific allele frequency differences, particularly the enrichment of risk haplotypes and depletion of protective alleles within the *MYCN*-amplified subgroup (Lim et al., 2024), is consistent with a model in which germline HLA configuration influences which tumor clones survive immune surveillance during early tumorigenesis, a process conceptually related to immune editing. However, this remains an inference from population-level allele frequency differences rather than direct evidence of clonal selection within individual tumors (e.g., from longitudinal immunopeptidomic or single-cell data) and should be presented as a hypothesis the field is positioned to test rather than an established mechanism.

**FIGURE 2:**
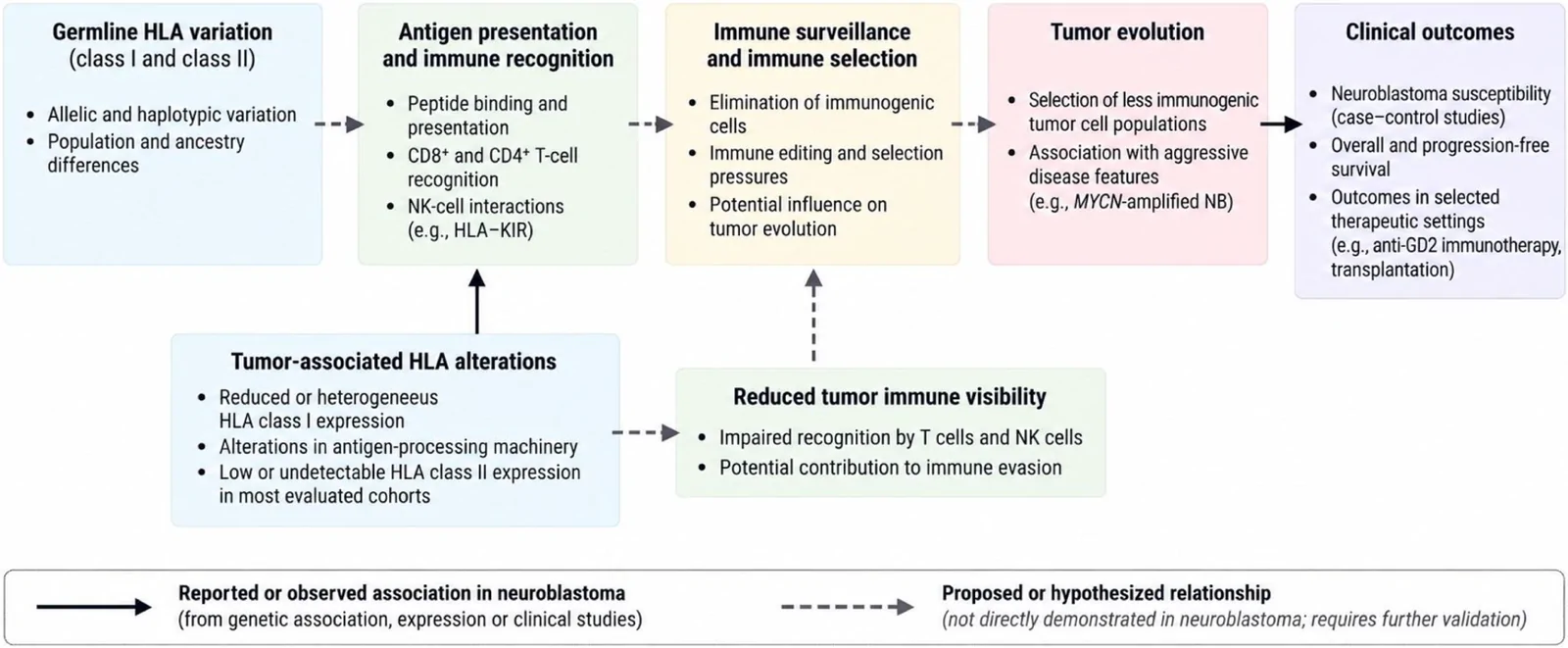
Proposed model linking germline HLA variation to NB development and clinical outcome. Inherited HLA class I and class II variation is associated with NB susceptibility and selected clinical outcomes. Potential effects of specific HLA configurations on antigen presentation, T-cell and NK-cell recognition, immune surveillance, and immune selection represent biologically plausible mechanisms that remain incompletely demonstrated in NB. Separately, tumor-associated alterations, including HLA reduced HLA expression and defects in antigen-processing pathways, have been observed in NB and may contribute to reduced tumor immune visibility and immune escape. Associations between HLA variation and tumor characteristics, including MYCN-amplified disease, do not establish a causal role for HLA in the development of these features. Solid arrows indicate reported or observed associations in neuroblastoma, whereas dashed arrows indicate proposed or hypothesized relationships. Created with BioRender.com and Adobe Illustrator.

The role of HLA in NB is multifaceted, involving both adaptive antigen presentation and innate immune regulation through KIR–NK-cell interactions. Inherited HLA variation may influence T-cell recognition, NK-cell responsiveness, and ultimately tumor immune surveillance. Distinct clinical associations were reported in specific therapeutic settings. HLA-Bw4-80I was associated with OS and PFS among patients receiving anti-GD2 immunotherapy (Forlenza et al., 2016), absence of the HLA-C1 ligand group with disease progression and survival following autologous HSCT (Venstrom et al., 2009), and higher PIRCHE-I/II mismatch scores with disease progression and survival following haploidentical transplantation (Seo et al., 2025). In contrast, A*02:01 and C*07:01 are associated with improved survival across disease stages, independent of treatment context (Fung and Pio, 2025). By contrast, the class II alleles associated with susceptibility (DQB1*05:02, DRB1*16:01) have not been consistently linked to more aggressive disease after onset, although a composite HLA genetic risk score has been associated with poorer OS (Bonfiglio et al., 2024).

This asymmetry, class II variation shaping susceptibility, class I variation shaping both susceptibility and post-diagnosis outcome, is itself a finding of the reviewed literature and may reflect the differing roles of CD4^+^ helper and CD8^+^/NK effector compartments at different stages of the tumor–immune relationship. Notably, clinical associations vary across outcomes. Evidence primarily supports associations with OS, PFS, and immunotherapy or transplantation outcomes, whereas evidence linking inherited HLA variation to chemotherapy response, treatment-independent relapse, or event-free survival remains limited.

### 5.2 Clinical and translational implications

Among reported clinical associations, the most consistent findings involve OS and PFS in defined immunotherapy or transplantation settings, whereas evidence concerning chemotherapy response or event-free survival remains limited. However, potential applications of germline HLA variation and treatment-specific immunogenetic parameters should be considered separately. Germline HLA alleles and haplotypes may eventually contribute to susceptibility or prognostic models, although modest effect sizes, limited replication, and population specificity currently preclude clinical application. By contrast, HLA–KIR ligand relationships, including Bw4 and HLA-C1, are relevant to NK-cell-mediated therapeutic settings (Forlenza et al., 2016; Venstrom et al., 2009), but treatment-by-genotype interactions establishing predictive benefit from anti-GD2 therapy remain unavailable. Similarly, donor–recipient mismatch measures, including PIRCHE-I/II scores, represent transplantation-specific parameters rather than germline susceptibility markers (Seo et al., 2025). As such, prospective evaluation of recipient and donor HLA/KIR genotypes and mismatch measures is needed before incorporating them into treatment-selection or risk-stratification strategies (Erbe et al., 2018).

Associations between HLA class I and II configurations and NB susceptibility also suggest the potential utility of HLA-informed risk models. However, the modest effect sizes reported to date, for example, odds ratios of approximately 1.6 for class II alleles (Bonfiglio et al., 2024), limit their use as standalone predictors. Integrating HLA with tumor characteristics, such as *MYCN* amplification status, may improve predictive performance, while the contributions of HLA heterozygosity and neoantigen presentation capacity remain to be determined in NB.

Notably, the largest case–control study to date found no significant associations between NB susceptibility and several classical or non-classical HLA class I loci at genome-wide significance thresholds (Bonfiglio et al., 2024). This underscores the modest magnitude of many reported effects and the need for independent replication. Discrepancies across studies likely reflect differences in cohort composition, HLA typing resolution, analytical approaches, and statistical power. Collectively, these findings support a role for host immunogenetic variation in NB susceptibility and progression and underscore the need for further studies to define how HLA diversity shapes immune surveillance in pediatric malignancies.

### 5.3 Gaps in evidence

Despite accumulating evidence linking HLA variation to NB susceptibility and clinical outcomes, several limitations constrain mechanistic interpretation and clinical translation. The relatively small size of most pediatric NB cohorts limits statistical power, reducing the ability to detect modest genetic effects and increasing uncertainty regarding negative findings and the reproducibility of reported associations. Most notably, the functional consequences of reported genetic associations remain insufficiently defined. Current studies are largely based on statistical associations and do not establish how specific alleles or haplotypes influence peptide presentation, T-cell selection, or broader antitumor immune responses. With the exception of structural inference for DQB1*05:02 (Bonfiglio et al., 2024), direct experimental validation is limited, underscoring the need for integrated immunogenetic and functional analyses.

Beyond individual alleles, metrics such as HLA evolutionary divergence and heterozygosity have emerged as important determinants of antitumor immunity in adult cancers (Bai et al., 2025). In broader immunogenetic and cancer studies, greater divergence between HLA alleles has been associated with broader peptide-binding repertoires and may enhance the range of presented tumor-derived antigens (Pierini and Lenz, 2018). However, these relationships have not been directly demonstrated in NB, and whether HLA diversity influences antigen presentation, immune surveillance, immune editing, or therapeutic response in NB remains uncertain and requires dedicated investigation.

At the tumor level, the relationship between germline HLA variation and HLA escape mechanisms remains poorly characterized, particularly whether inherited HLA alleles or haplotypes influence the emergence of tumor-associated HLA downregulation, loss of heterozygosity, or antigen-processing defects in NB. In addition, key immunogenetic parameters, including HLA heterozygosity, evolutionary divergence, and breadth of peptide-binding repertoire, as well as potential interactions with KIR variation, have not been systematically evaluated in NB, despite their established relevance in adult malignancies. Incomplete consideration of NB heterogeneity compounds the interpretation of the associations. While some studies examined *MYCN* amplification, most did not systematically assess whether HLA associations varied by stage, risk classification, age, or other molecular features. Since these factors strongly shape tumor biology, their neglect may explain inconsistent findings and obscure subgroup-specific effects.

Finally, prospective clinical evidence is lacking. Although retrospective studies have associated immunogenetic variation with survival and disease-progression outcomes among patients receiving anti-GD2 immunotherapy or undergoing transplantation, this does not distinguish prognostic effects from treatment-specific predictive effects. It remains unclear whether HLA/KIR profiling improves prognostic accuracy or identifies patients who benefit differentially from a particular therapy. In addition, the role of HLA-KIR interactions in NB remains to be further addressed, and future studies integrating high-resolution HLA typing, KIR genotyping, NK-cell phenotyping, and clinical outcome data are warranted to clarify how inherited immunogenetic variation influences tumor susceptibility, immune surveillance, and response to immunotherapy. Addressing these gaps will require coordinated, multidisciplinary studies that integrate genetic, functional, and clinical data.

### 5.4 Limitations

Several limitations affect the interpretation and generalizability of the available evidence. A key limitation is the lack of independent replication. Aside from the chromosome 6p22 signal, most classical HLA associations are based on single cohorts or re-analyses of existing datasets, with subgroup recurrences reflecting internal consistency rather than independent validation. A marked population bias is evident, as most studies, including the largest datasets, are derived from European or North American cohorts (Bonfiglio et al., 2024; Lim et al., 2024), limiting applicability to populations with distinct HLA architectures. Notably, the only cross-population replication at 6p22 identified a different signal (He et al., 2017), underscoring potential population-specific effects.

Statistical power is further constrained by limited sample sizes, ranging from fewer than 50 cases in early studies to approximately 724–803 cases in the largest recent cohorts (Bonfiglio et al., 2024), as well as inconsistent approaches to multiple-testing correction. For example, early associations such as HLA-B13 did not remain significant after adjustment (Abrahámová and Májský, 1988). These cohort sizes remain modest for highly polymorphic loci such as HLA genes, where large numbers of alleles and haplotypes are tested simultaneously, increasing the likelihood of both false-negative findings and inconsistent replication across studies. Comparability across studies is further hindered by heterogeneity in HLA typing methods, ranging from low-resolution serology to high-resolution sequencing (Lim et al., 2024), which may contribute to inconsistent replication.

The focus on NB also precludes broader inference to other pediatric solid tumors, leaving the general relevance of proposed immunogenetic mechanisms unresolved. Furthermore, most reported associations lack functional validation, except for structural inference for DQB1*05:02 (Bonfiglio et al., 2024), highlighting a gap between genetic association and immunological mechanism. The study cohort’s limited ancestral diversity is another concern, given that HLA allele frequencies vary substantially across populations, limiting the generalizability of these findings. Future studies should validate these associations in diverse, multicenter cohorts using high-resolution HLA genotyping to better capture population-specific variation and assess the consistency of observed effects across different ancestral backgrounds. Finally, fragmented study designs, in which susceptibility and clinical outcomes are typically assessed separately, limit comprehensive evaluation of allele and haplotype effects across the disease continuum.

## 6 Conclusions and future directions

Accumulating evidence associates inherited variation at classical HLA class I and class II loci in NB susceptibility and suggests associations with clinical outcomes in selected therapeutic settings. Effects on antigen presentation and immune surveillance provide biologically plausible mechanisms for these associations, but remain incompletely validated experimentally in NB (Figure 2). Of the loci evaluated to date, the strongest evidence supports involvement of *HLA-A*, *HLA-B*, and *HLA-C* class I loci and the *DRB1-DQB1* class II interval in shaping NB susceptibility and clinical behavior. Tumor-associated HLA alterations, including reduced HLA expression and defects in antigen presentation, constitute distinct mechanisms of immune evasion that may contribute to disease progression and treatment resistance. The most consistent associations map to extended haplotypes rather than individual alleles. Whether similar immunogenetic mechanisms operate in other pediatric solid tumors, including Wilms tumor, hepatoblastoma, and sarcomas, remains unclear and will require high-resolution, adequately powered genotyping studies comparable to those recently conducted in NB (Bonfiglio et al., 2024; Lim et al., 2024).

Three priorities emerge. First, independent replication of HLA-A*02:01∼B*44:02∼C*05:01, B*07:02, DQB1*05:02, DRB1*16:01, and DRB1*15:01∼DQA1*01:02∼DQB1*06:02 associations in additional, ancestrally diverse NB cohorts is necessary to establish generalizability. Second, integration of high-resolution HLA typing with functional immunological approaches, including neoantigen prediction, measures of HLA heterozygosity, and peptide-binding assays, would clarify whether the reported genetic associations translate into differences in tumor immunogenicity in NB, an issue that currently remains largely hypothetical. Third, prospective studies examining HLA-KIR interactions and NK-cell biology, and evaluating the combined effects of HLA and KIR genotypes on outcomes following anti-GD2 immunotherapy and haploidentical transplantation, would extend existing retrospective observations (Forlenza et al., 2016; Seo et al., 2025; Venstrom et al., 2009) and inform the potential clinical application of these findings in treatment selection for high-risk NB.

Together, these directions support a transition from descriptive genetic associations to clinically relevant models for risk stratification and immunotherapy optimization in pediatric NB, with potential extension to other pediatric solid tumors. Current evidence suggests that the clinical relevance of HLA variation is most apparent for survival-related endpoints and clinical outcomes observed in immune-based treatment settings, including among patients receiving anti-GD2 immunotherapy and undergoing hematopoietic stem cell transplantation, rather than for responses to conventional chemotherapy. While this underscores the importance of HLA-mediated immune mechanisms in neuroblastoma biology, these immunogenetic markers remain hypothesis-generating rather than clinically actionable, and the available evidence does not establish HLA/KIR characteristics as treatment-specific predictive biomarkers. Validation in larger, multicenter cohorts with diverse ancestral representation and high-resolution HLA genotyping will be important to establish the generalizability of these findings.
